# Tea Polyphenols as Prospective Natural Attenuators of Brain Aging

**DOI:** 10.3390/nu14153012

**Published:** 2022-07-22

**Authors:** Mengyu Hong, Jing Yu, Xuanpeng Wang, Yanan Liu, Shengnan Zhan, Zufang Wu, Xin Zhang

**Affiliations:** 1 Department of Food Science and Engineering, Ningbo University, Ningbo 315211, China; a13056961758@163.com (M.H.); liuyanan@nbu.edu.cn (Y.L.); zhanshengnan@nbu.edu.cn (S.Z.); zxdqqzyc@163.com (Z.W.); 2 Guangdong Qingyunshan Pharmaceutical Co., Ltd., Shaoguan 512699, China; yujing5249@foxmail.com

**Keywords:** tea polyphenols, intestinal microbiota, anti-aging, microglia, neurological diseases

## Abstract

No organism can avoid the process of aging, which is often accompanied by chronic disease. The process of biological aging is driven by a series of interrelated mechanisms through different signal pathways, including oxidative stress, inflammatory states, autophagy and others. In addition, the intestinal microbiota play a key role in regulating oxidative stress of microglia, maintaining homeostasis of microglia and alleviating age-related diseases. Tea polyphenols can effectively regulate the composition of the intestinal microbiota. In recent years, the potential anti-aging benefits of tea polyphenols have attracted increasing attention because they can inhibit neuroinflammation and prevent degenerative effects in the brain. The interaction between human neurological function and the gut microbiota suggests that intervention with tea polyphenols is a possible way to alleviate brain-aging. Studies have been undertaken into the possible mechanisms underpinning the preventative effect of tea polyphenols on brain-aging mediated by the intestinal microbiota. Tea polyphenols may be regarded as potential neuroprotective substances which can act with high efficiency and low toxicity.

## 1. Introduction

With increase in life expectancy and decrease in the birth rate, the age of the global population is increasing exponentially. Age-related neurodegenerative diseases are one of the main causes of morbidity and mortality in contemporary society. They create a huge global health and social burden. The United Nations predicted that the proportion of the world’s population over the age of 60 will double from 962 million to 2.1 billion by 2050 [1]. With aging, the structure and neurophysiology of the human brain show varying degrees of decline. Oxidative stress, mitochondrial dysfunction, increased inflammatory response, imbalance of energy metabolism, impairment of DNA repair function, decrease in neurotrophic factors, cellular senescence and telomere depletion are considered risk factors for neurodegenerative conditions, such as Parkinson’s disease (PD), Alzheimer’s disease (AD) and Huntington’s disease (HD) [2]. Senescence is usually caused by chronic effects, including telomere shortening, DNA damage and carcinogenic signal transduction. As an important sign of aging, the length of telomeres at the end of chromosomes gradually shortens until they completely disappear, resulting in no further cell division. Senescent cells have three main characteristics: blocked cell proliferation, a complex senescence-associated secretory phenotype and anti-apoptosis [3]. In the process of aging, repeated antigen stimulation creates continuous pressure on the immune system, leading to an increase in the secretion of pro-inflammatory cytokines, such as tumor necrosis factor (TNF)-α and interleukin (IL)-6. These pro-inflammatory factors may generate an inflammatory immune system state, eventually leading to a decline in age-related adaptive immunity, including altered responses of B and T cells [4].

The intestinal tract is a key target organ for improving the health of elderly animals and human beings. Some studies have considered the role of the intestinal microflora in the treatment of senile neurodegenerative diseases [5,6]. With age, the digestive and absorptive capacity of the gut weakens, intestinal stem cells are depleted and their activity decreases, resulting in a reduction in the size and density of intestinal villi [7]. Microglia are resident immune cells in the central nervous system, which are closely linked to central nervous system diseases, such as AD, PD and multiple sclerosis. The intestinal microflora is considered to control the maturation and function of microglia [8].

In addition to antioxidant and anti-inflammatory functions, plant polyphenols also protect nerve cells by regulating the signal cascades of a series of protein kinases and lipid kinases [9]. The processing of green tea includes steaming or baking to inactivate polyphenol oxidase and achieve enhanced antioxidant effects. The content of tea polyphenols (TP) in green tea is therefore particularly high [10]. TP act synergistically with the intestinal microflora to reduce oxidative stress, inhibit inflammation, upregulate nuclear factor-erythroid 2 p45-related factor2 (Nrf2), protect the intestinal barrier and alleviate the symptoms of brain aging [11]. The intestinal microbiota metabolize TP into microbial metabolites which have a higher absorption rate and greater biological activity than the precursor polyphenols. TP increases the diversity of the intestinal microbiota, improves the number of beneficial microflora and inhibits some pathogenic strains. This interaction between TP and the intestinal microbiota contributes to the relief and alleviation of intestinal-related diseases [11,12,13]. TP can also be decomposed into different metabolites by microflora in the colon, many of which have higher anti-inflammatory activity and bioavailability than their precursors [11,12,14]. The structural characteristics of the polyphenol carbon ring, and the number of hydroxyl groups on the ring, are the main determinants of prolonged metabolic activity [15,16]. In aging and age-related neurodegenerative diseases, TP not only inhibit apoptosis signals in mitochondria, but also protect nerve cells by enhancing the activity of neurotrophic factors [17,18]. This paper reviews several factors that may induce brain aging, the role of the intestinal microflora on the intracellular homeostasis of microglia and the molecular mechanisms of the neuroprotective effect of TP during aging.

## 2. Possible Triggers and Mechanisms of Cognitive Decline in the Process of Brain Aging

A key early finding in the field of aging research was the observation in 1939 that limiting food intake in mice and rats could prolong life [19]. The thinning of the cerebral cortex is related to a decline in cognitive ability and AD. Because the left and right hemispheres are responsible for different functions, their cortical thickness is also different; this phenomenon is called cortical asymmetry. The researchers found that the asymmetry of the cerebral cortex disappeared with age, indicating that the rate of degeneration was different on opposite sides of the brain. In equivalent brain areas, it was found that the left side of the brain shrank faster in patients with AD [20]. Previous studies have attributed the neuronal damage observed in AD to what happens following the accumulation of β-amyloid (Aβ) outside the cell, rather than before it and inside the neuron. Recent studies have found that the neuronal damage observed in AD may be attributed to the lower activity of lysosomal acidic enzymes in brain cells; before plaques are formed outside the cells, the nerve cells are paralyzed [21].

### 2.1. Dysregulation of Neuronal Calcium Homeostasis

The cellular activities of nerve cells are affected by a variety of substances. Nerve cells transmit and integrate signals through the flow of various ions, neurotransmitters and regulators between the presynaptic and postsynaptic membranes, to effect a variety of brain functions. The concentration of various substances inside and outside the membrane of brain cells, including ion concentration, is particularly important for the normal functioning of brain cells [22]. Neurons use calcium signals to control membrane excitability, neurotransmitter release, gene expression and the neuronal cell cycle. These cells are particularly vulnerable to interference of Ca^2+^ homeostasis [23]. In the process of aging, the ability of neurons to control the dynamic balance of Ca^2+^ in the normal range is impaired [24,25]. Ca^2+^ controls the level of reactive oxygen species (ROS), not only by regulating the activity of mitochondria, but also by affecting the activity of nicotinamide adenine dinucleotide phosphate oxidase (NOX) enzymes [26]. Both NOX1 and NOX2 are abundantly expressed in the central nervous system, and NOX2 is significantly upregulated in animal models with neurodegenerative diseases, including AD, HD, PD and multiple sclerosis [27]. In addition, it has been found that ROS and Aβ produced by NOX2 are more likely to be deposited in the brain tissue of aged mice. Aβ, which is associated with AD, has been found to promote cellular calcium overload by reducing the integrity of the plasma membrane through oxidative stress [28]. The activity of plasma membrane Ca^2+^-ATPase (PMCA) is also related to age and oxidation. The impaired activity and decreased expression of PMCA can disrupt cellular calcium homeostasis, leading to cytotoxic calcium overload and calcium-dependent cell death, including apoptosis and necrosis. It may also impair neuronal function in the brain and increase susceptibility to neurodegenerative diseases during aging [29,30]. In addition, the depletion of calcium homeostasis modulator 1, a calcium channel involved in the regulation of intracellular Ca^2+^ levels, was found to downregulate the production of ROS, activate the expression of hypoxia inducible factor-1α, and show neuroprotective effects [31].

### 2.2. Mitochondrial Oxidative Stress in Brain Cells

The mitochondria regulate cell function, not only by producing energy, but also by generating ROS. Excessive ROS can damage mitochondria by inducing mitochondrial DNA mutations, changing mitochondrial membrane permeability, disrupting calcium homeostasis and destroying respiratory chains, resulting in mitochondrial and cellular dysfunction [32]. Through the opening of mitochondrial permeability transition pores (MPTPs), mitochondria can release cytochrome C, apoptosis-inducing factor and Ca^2+^ into the cytoplasm, destroying the overall structure of cells through a variety of mechanisms, causing functional disorders, and eventually leading to apoptosis [33]. When mitochondria are damaged or malfunctioning, less ATP is produced and less energy is supplied for the brain to function properly [34]. The brain is the most important organ of the human body, accounting for only 2–3% of the body’s mass but consuming 20% of the oxygen and 25% of the glucose [35,36]. Therefore, compared with other organs, the brain is more sensitive to changes in energy supply and is more vulnerable to oxidative stress.

Brain aging can activate the pathological pathways of AD. In contrast to normal aging, AD is accompanied by progressive slowing of cognitive processes, synaptic degeneration, deficiency in axonal transport, and increased expression of apoptotic enzymes in active forms, such as caspase-3 and pro-caspase-3 [37,38]. The decline in mitochondrial function during brain aging decreases intracellular nicotinamide adenine dinucleotide (NAD^+^) levels and the NAD^+^/NADH ratio [39]. This may impair the activity of NAD^+^-dependent enzymes that are critical to neuronal functioning and survival, including sirtuin family protein deacetylases [40]. NAD^+^ is a key longevity factor, which has a significant impact on the determinants of aging, including mitochondrial homeostasis, oxidative damage, calcium homeostasis, neural networking, DNA repair and inflammation. Therefore, targeting NAD^+^ metabolism has become a potential treatment to improve aging-related diseases and prolong human life [41,42,43]. It has been found that mitochondria are the main targets for oxidative damage in middle-aged and elderly mice [44]. Oxidative stress markers, such as protein carbonation, lipid oxidation and mitochondrial DNA oxidation are increased in the aging brain [45,46]. TP protects nerve cells by inhibiting or inducing the opening of MPTPs and apoptosis in vivo and in vitro.

### 2.3. Telomere Shortening and Telomerase Dysfunction

**Figure 1:**
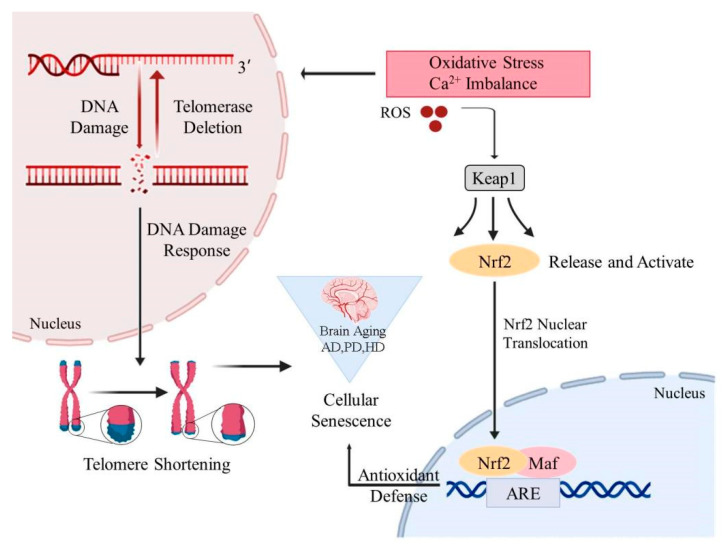
Possible triggers of brain aging. Oxidative stress and Ca^2+^ imbalance can lead to increased ROS levels, excessive ROS will cause DNA mutation, telomere disorders trigger age-related diseases. Excessive ROS also activate the transfer of Nrf2 to the nucleus which binds to Maf protein to form heterodimers. ARE in the form of Nrf2-Maf carry out antioxidant defense against age-related brain diseases. ROS = reactive oxygen species; Keap1 = Kelch-like ECH-associated protein 1; Nrf2 = nuclear factor-erythroid 2 p45-related factor2; ARE = antioxidant response elements.

The telomere is a unique DNA-protein structure at the end of chromosomes, which can protect chromosomes from damage. Telomerase is a ribonucleoprotease used to maintain telomere length [47]. With age, the telomeres in brain cells become shorter. The telomeres of microglia in the cerebellum and cortex of aged rats were significantly shorter than those of young rats [48]. Telomerase knockout mice are associated with neuronal loss in the hippocampus and frontal cortex, impaired spatial learning and memory, while telomerase reactivation delays and reverses many cognitive performance declines associated with aging [49]. Repeated DNA replication in the absence of telomerase leads to telomere dysfunction and shortening, which can lead to senescence that limits cell proliferation [50]. Dysfunctional telomeres in microglia and peripheral immune cells trigger a persistent DNA damage response, which, in turn, leads to cell cycle arrest and the expression of pro-inflammatory factors related to aging-related secretory phenotypes [51]. Chronic obstructive pulmonary disease, neurodegenerative diseases, inflammatory bowel disease and other diseases are more likely to occur. The most popular hypothesis is that it is not telomere dysfunction itself that causes aging and aging-related diseases, but the DNA damage response activated by telomere dysfunction that leads to cell aging [52]. DNA damage can cause protein homeostasis stress, and the protein homeostasis pathway controls protein synthesis, folding and degradation. Many age-related diseases, including AD and PD, are closely related to misfolding and aggregation of proteins [53]. Overall, these observations suggest that the stabilization and maintenance of telomeres and telomerase are necessary for brain function [54] (Figure 1).

## 3. TP Suppress Brain Aging-Related Signaling Pathways and Hallmarks

Morphologically, the main features of brain aging are a reduction in brain volume, thinning of the cortex, degradation of white matter and gray matter, decrease in cerebral gyruses and the enlargement of ventricles. In pathophysiology, brain aging is related to nerve cell atrophy, dendritic degeneration, vascular disease, slow metabolism, activation of microglia and formation of white matter lesions [55]. The rate of brain atrophy during aging can predict whether a person will develop cognitive impairment and dementia. The hippocampal volume of clinically diagnosed AD patients was 15–40% smaller than that of healthy controls [56]. The annual hippocampal atrophy rate of AD patients was 4.66%, and that of healthy controls was 1.41% [23,56].

Natural TP are cellular redox regulators, which may help to reduce the incidence of some pathological diseases, including metabolic diseases, cardiovascular and neurodegenerative diseases, and which may have anti-aging properties [57,58,59]. The proportion of flavanols in TP is high, with catechin being the most important, accounting for approximately 60–80% of the total polyphenols. Catechin includes two galloylated polyphenols (−)-epigallocatechin-3-gallate (EGCG, 59%) and (−)-epicatechin-3-gallate (ECG, 13.6%), and two nongalloylated polyphenols (−)-epigallocatechin (EGC, 19%) and (−)-epicatechin (EC, 6.4%) [57].

Oxidative stress-induced ROS production and inflammation play a key role in inducing neurodegenerative diseases. TP can scavenge ROS, induce endogenous antioxidant enzymes, and chelate excess bivalent metals, such as iron and copper [60,61]. The neuroprotective effect of TP has been confirmed in a number of studies, which have shown that long-term tea drinking is negatively correlated with the incidence of dementia, AD and PD. This may explain why the incidence of age-related neurological diseases is lower in Asians than in Europeans or Americans [62,63]. In human neuroblastoma SH-SY5Y cells, EGCG can prevent nerve cell death induced by neurotoxins 6-hydroxydopamine and 1-methyl-4-phenylpyridinium [64]. In another study, EGCG was found to inhibit the activation of extracellular signal-regulated kinase cascade (ERK) and NF-kB in the brain of Aβ_1–42_ injected mice, thereby increasing the activity of α-secretase, inhibiting the activity of β-and γ-secretase, and improving cognitive impairment. The above findings support the possibility of the use of TP as a dietary supplement in the prevention of neurodegenerative diseases [65].

Long-term consumption of tea, especially black or oolong tea, is associated with reduced risk of cognitive impairment [66]. Among 2031 elderly Norwegian people, a tea drinking group scored significantly higher on cognitive tests than a non-tea drinking group [67]. In the United States, people who drink two or more cups of tea a day were found to have a lower risk of Parkinson’s disease [68]. A single dose of EGCG can change several parameters of human cerebral blood flow, and a lower dose of EGCG (135 mg) can induce a decrease in cerebral blood flow [69]. In a recent study, 36 subjects were assigned to take shentai TP for 90 days. The results showed that supplementation of shentai TP for three months could improve the compensatory response and cognitive reserves of the brain [70].

An increase in ROS is not always harmful to cells. Some studies have shown that a transient increase in ROS may protect cells by inducing transcriptional changes in the nucleus via a mitohormetic response and participating in potential feedback mechanisms of antioxidant defense or stress defense pathways, such as the Nrf2 signal pathway [71]. In this mitohormetic response, EGCG and ECG in tea catechins initially act as pro-oxidants which activate an antioxidant defense mechanism by stimulating a transient increase in ROS. The level of ROS increased significantly after 6 h EGCG treatment and 12 h ECG treatment but decreased significantly after 24 h and 120 h treatment [72]. EGCG and ECG, at a concentration of 2.5 μM, was able to enhance the lifespan of *Caenorhabditis elegans* [73]. If the concentration of EGCG and ECG is too high, it may produce excessive ROS and show harmful effects [73,74]. When mice were given EGCG 1500 mg/kg by intragastric administration, it was found that the glutamic pyruvic transaminase activity increased 138-fold, the hepatotoxicity increased, and the survival rate decreased by 85%. It has been suggested that high doses of EGCG have a toxic effect on the liver, which may be related to the pro-oxidative activity of EGCG [75]. After administration of 50 μM EGCG, the level of hydrogen peroxide increased, and the level of glutathione decreased in a dose-and time-dependent manner [72]. However, the mechanism of ROS formation induced by EGCG and ECG is not clear [73]. Excessive ROS can destroy cell proteins, lipids and DNA, resulting in fatal cell damage, which, in turn, involves a variety of pathology, such as aging, cancer, neurodegenerative diseases, cardiovascular disease, diabetes, and others [76,77]. The neuroprotective effects of TP are shown in Table 1.

### 3.1. TP Increase the Expression of Neurotrophic Factors and Their Receptors

Neurotrophic factors are a family of proteins that induce neuronal survival, development and function. Neurotrophic factors mainly include brain-derived neurotrophic factors (BDNF), nerve growth factor (NGF), and neurotropin-3 (NT-3) and neurotropin-4 (NT-4). These proteins are potential drug targets for the treatment of nerve injury and other diseases. Neurotrophic factors have two different membrane protein receptors, p75 neurotrophin receptor (p75^NTR^) and tropomyosin-related kinase (Trk) receptors, consisting of three receptors, TrkA, TrkB, and TrkC, in mammals [82]. The activation of the Trk receptor promotes neuronal survival, while the activation of p75^NTR^ leads to cell death. By interacting with the extracellular regions of these two receptors, neurotrophic factors transmit signals about nerve cell survival and apoptosis to the inside of the cell, thus regulating cell development and apoptosis [2].

A decrease in the functioning of neurotrophic factors and their receptors can lead to neuronal damage and contribute to the pathogenesis of neurodegenerative diseases [83,84]. Different neurotrophic factors have different receptors. NGF binds more easily to TrkA receptors [85,86]. TrkB is a high affinity receptor for BDNF and NT-4, and NT-3 is the main ligand of the TrkC receptor [85]. EGCG played a neuroprotective role in a rat pheochromocytoma cell line, PC12. PC12 cells exhibited ectopic expression of TrkB and induction of neurite growth to enhance BDNF [80]. It has been found that a low concentration (2 ng/mL) of BDNF has little effect on the neurite growth of PC12 TrkB cells [80]. TP may be suitable for the long-term treatment of nervous system diseases. Green tea polyphenols (GTP) (0.05–5 μg/mL) alone could not induce cell neurite growth. When GTP were at a concentration of 0.05–0.2 μg/mL combined with a low concentration of BDNF, the neural differentiation ability of BDNF was significantly enhanced. This implies that GTP in combination with BDNF can better promote neurite outgrowth [80]. Studies have shown that a low concentration of GTP (0.1 μg/mL) can promote axonal growth induced by a low concentration of BDNF (2 ng/mL). This promotional effect was equivalent to that of a high concentration of BDNF (50 ng/mL) [80]. After oral administration of 800 mg EGCG, the content of EGCG in plasma was as high as 0.4–0.8 μM, which was found to be safe for humans [87]. Because of its hydrophilicity, EGCG cannot cross the blood-brain barrier efficiently. In one study, only 0.5 nmol/g of EGCG reached the brain after 60 min of a single oral administration of GTP (500 mg/kg body weight) [80]. It has been estimated that a cup of tea (2.5 g green tea soaked in 200 mL of water) may contain 90 mg of EGCG [88]. In general, human intake of polyphenols ranges from 100 mg/d to 1 g/d [89]. The experimental amount of TP used in animals is much higher than the actual concentration of TP in human organs and blood after drinking tea [90]. The concentration of TP used in experiments is generally between 20 and 100 μg/mL, while the concentration of TP in human blood after drinking tea is usually approximately 0.5 μg/mL [91]. The optimal concentration of GTP to enhance BDNF-induced neurite growth is 0.1 μg/mL [80]. Therefore, after drinking green tea, this concentration in the body is sufficient to enhance the effect of BDNF.

EGCG has also been found to enhance the effect of NGF on nerve regeneration, when followed by ECG. EC and EGC alone cannot enhance the neurite growth induced by NGF but can be used in combination with EGCG to promote the axonal growth activity of PC12 cells [92]. The combination of GTP and NGF not only increases the number of neurites, but also increases the length and branching degree of neurites [82,93]. This shows that there is a synergistic effect among the mixed extracts of TP—the mixture may provide a stronger effect than the individual components [92,94].

### 3.2. TP Activate Signal Pathways for Neurotrophic Function

In several tissues of human and mice, the transactivation of nuclear factor-κB (NF-κB) is closely related to aging. Oxidative stress does not directly activate the NF-κB complex but uses ROS to target redox-sensitive protein phosphatases and protein kinases, thus activating NF-κB signal transduction [95]. The function of NF-κB has two aspects. It is the normal physiological need of the body to appropriately activate the adaptive stress response program mediated by NF-κB and to support the survival of neurons by increasing the expression of antioxidants, growth factors and anti-apoptotic molecules [96]. However, overactivation increases the activity of NF-κB, induces neurotoxicity, and promotes neuronal death by inducing the production of pro-inflammatory cytokines, such as IL-6, TNF-α and cyclooxygenase-2 [96]. Studies have shown that chronic low-level inflammation throughout the body can accelerate the aging process [97,98]. Upstream inhibitors of NF-κB signal, AMP-activated protein kinase (AMPK) and silent information regulator 1 (SIRT1) are well-known anti-inflammatory factors and have beneficial effects on health and longevity [99]. The two main molecular targets of TP, the Nrf2 and AMPK signal pathways, can inhibit oxidative stress [100]. TP can activate SIRT1, the downstream target of AMPK [73]. SIRT1 can directly act on NF-κB and reduce the acetylation level of the p65 subunit Lys^310^, inhibit its transcriptional activity and downregulate the expression of pro-inflammatory genes [101]. Knockout of SIRT1 can lead to excessive acetylation of NF-κB and increased inflammatory response [102]. Recent studies have shown that polyphenols are powerful antioxidants as free radical scavengers in vitro, but they can be used as pro-oxidants at high doses. Therefore, TP should be used appropriately [103]. Catechin and EGCG monomers blocked the DNA binding of NF-κB in the late stage, resulting in inactivation of the NF-κB pathway [104]. Polyphenol monomers can significantly inhibit the NF-κB cascade pathway at different stages due to their different chemical structures [104,105].

Nrf2, as a key transcription factor regulating antioxidant stress, plays an important role in inducing an antioxidant response, such as regulation of redox balance, cell proliferation and autophagy, proteasome degradation, DNA repair and mitochondrial physiological function [106]. Kelch-like ECH-associated protein 1 (Keap1) is one of the main regulators of intracellular Nrf2 level and exists in the cytoplasm in an inactive state where it binds to Nrf2 under normal conditions [107]. When cells are exposed to oxidative stress, Nrf2 dissociates from Keap1 and is activated, translocates to the nucleus, and binds to the Maf protein to form a heterodimer. It then combines to antioxidant response elements (ARE), which initiates downstream gene expression in the form of Nrf2-Maf, thus further activating gene expression of detoxification and antioxidant defense mechanisms [108] (Figure 1). Nrf2 is ubiquitous in tissues, especially in the central nervous system, promoting cell survival and coordinating the transcription of neuroprotective proteins, and is a major anti-aging regulator [100]. Compared with a normal group, Nrf2 knockout mice showed increased gliosis and dopaminergic nigral striatal degeneration [109]. Activating Nrf2 signals can combat oxidative damage in age-related diseases, especially in chronic inflammatory conditions, thus increasing healthy life expectancy [110]. EGCG was an effective neuroprotective agent in an AD model. EGCG increased the important endogenous antioxidants in microglia [111]. EGCG inhibited β-induced cytotoxicity by reducing the activation of the mitogen-activated protein kinase (MAPK) signal transduction pathway [112,113]. Studies have shown that EGCG can activate the expression of heme oxygenase 1 through the ARE/Nrf2 pathway and protect neurons from oxidative damage [72,114].

MAPK is an important signal transducer from the cell surface to the interior of the nucleus. The neuroprotective mechanism of EGCG may depend on the regulation of MAPK and its downstream pathway. The MAPK family is composed of p38 MAPK cascades, c-Jun NH2-terminal kinases (JNK) and ERK, which are closely related to neurodegenerative diseases [115]. The function of JNK is similar to that of p38, which is related to inflammation, apoptosis and growth [116,117]. ERK mainly controls cell growth and differentiation. Oxidative stress induced by ROS was found to activate both p38 and JNK, and the level of phosphorylation increased [118]. GTP at a concentration of 4 μg/mL downregulated the expression of the caveolin-1 gene by activating ERK1/2 and inhibiting the p38 signal transduction pathway to regulate a variety of cell activities, such as cell proliferation, apoptosis and inflammation [119]. At a concentration of 10 μM in vitro, EGCG was found to inhibit the phosphorylation of ERK1/2 by interfering with the binding of the protein substrate to protein kinase [119]. Studies have shown that EGCG can maintain cell vitality, reduce the level of ROS, increase the expression of BDNF, regulate the apoptosis of mitochondrial pathway and MAPK pathway, and thus improve brain aging and prevent neurodegenerative diseases [120].

## 4. Crosstalk between Brain and Gut Microbiome Have Certain Impact on Aging

**Figure 2:**
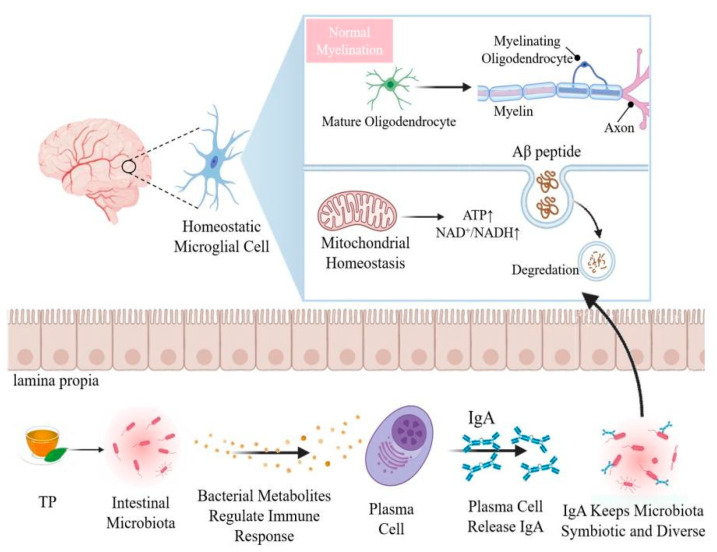
Intestinal flora and metabolites bind to IgA to maintain homeostasis of cerebral microglia. After ingestion of TP, most are metabolized into small molecules by microbes in the colon, which regulate the immune response. IgA is produced by plasma cells in the intestinal tract. The presence of IgA enriches the quantity and variety of intestinal flora. The intestinal microbiota is very important to maintain the functional state of microglia, and microglia are key immune cells to prevent neurodevelopmental abnormalities and neurodegenerative diseases. IgA = immunoglobulin A; NAD^+^ = nicotinamide adenine dinucleotide; Aβ = β-amyloid.

The intestinal microflora is considered essential for key physiological processes of the brain, such as myelin formation, neurogenesis and microglial activation [121]. The host microflora affect the maturation of microglia through metabolite single-chain fatty acids and regulate the activity of astrocytes through tryptophan and aromatic hydrocarbon receptors [122]. The intestinal microflora is very important for maintaining the functional state of microglia, and microglia are key immune cells in the prevention of neurodevelopmental abnormalities and neurodegenerative diseases [123]. The expression of age-related genes did not increase in aging germ-free mice, indicating that the microflora determines the activation of microglia and plays a key role in regulating the immune response, cytokine production and toll-like receptor (TLR) signals of aged microglia [124]. The integrity of the blood-brain barrier in the frontal cortex, hippocampus and striatum of rats is affected by the intestinal microflora [125]. The meninges are layered units of membranous connective tissue that cover the brain and spinal cord. Mouse and human meninges contain plasma cells that secrete immunoglobulin A (IgA). B cell receptor sequencing confirmed that meningeal IgA^+^ cells originate from the intestinal tract [126]. In the mucosa-associated lymphoid tissue of the gut, specific signals lead to the development of plasma cells that produce IgA [126]. Researchers have found that IgA plasma cells, which originate in the gut, protect the brain from pathogens [127]. Compared with normal mice, there was almost no IgA in the meninges of aseptic mice, and the meningeal IgA network was completely restored after the intestinal tract of these mice was reconstructed with microorganisms [128]. This shows that intestinal bacteria are very important for the existence of meningeal IgA cells, and the healthy and steady state of the intestinal flora directly affects the protection of the brain against inflammation (Figure 2).

Recently, it has been found that hypothalamic neurons can directly sense the structural components of the intestinal bacterial microbiota and control food intake and body temperature [129]. The researchers knocked out the Nod2 of inhibitory γ-aminobutyric acid neurons in mice and found that peptidoglycan derivative cell wall peptides no longer exerted an inhibitory effect. The mice gained weight faster and lived for a significantly shorter period than the normal group. Cells can respond to bacterial cell wall peptides, which means that fluctuation in the bacterial population in the intestinal tract has a direct regulatory effect on the activity of neurons [129,130]. A recent study investigated the effects of aged intestinal microflora on the cognitive ability of young rats by transplanting fecal microbiota of old rats with cognitive impairment [131]. The results showed that bacterial transplantation impaired the cognitive behavior of young recipient rats, causing decreased homogeneity of the medial prefrontal cortex and hippocampus, and impaired the functional activity of neurons in BDNF and specific brain regions of rats. In addition, young rats showed enhanced levels of oxidative stress and pro-inflammatory cytokines after flora transplantation, suggesting that intestinal oxidative stress and inflammation may be reasons for the decline in cognitive function during aging [18,132,133]. When the feces of young mice were transplanted into aged mice, the differences in peripheral immunity, brain immunity, hippocampal metabolism group and transcriptional group were reversed. In addition, the microbiota from young donors was able to reduce the selective impairment of age-related cognitive behavior, thus confirming that the microbiome may be a suitable therapeutic target for promoting healthy aging [134].

## 5. Effects of TP on Brain Aging by Modulating Gut Microbiota

Cumulative studies have shown that the absorption rate of most polyphenols in the small intestine is very low [135,136]. Some free and simple polyphenols are degraded by lactase phloridzin hydrolase at the brush edge of intestinal epithelial cells, and the resulting aglycones enter colon cells in a passive manner [137,138]. There are about 10^12^ kinds of microorganism in the colon, and the residual polyphenols and aglycones undergo ring division under the action of microorganisms [139]. After arriving in the colon, catechin was first converted by *Eggerthella lenta* into 1-(3,4-dihydroxyphenyl)-3-(2,4,6-trihydroxyphenyl) propan-2-ol (2,4,6-THPL), and then converted by *Flavonifractor plautii* into 5-(3,4-dihydroxyphenyl)-γ-valerolactones (3,4-DHPL) and 4-hydroxy-5-(3,4-hydroxyphenyl)valeric acid (3,4-HPVA) [139]. As the most abundant metabolite of catechins, 3,4-DHPL has stronger anti-adhesion and antioxidant activities than the catechin parent compounds. The mechanism involves the downregulation of NF-κB transcription by phosphorylation of IKK and IκB. After drinking green tea, microbial metabolites of 3,4-dihydroxyphenylacetic acid (3,4-DHPAA) were also detected [139,140]. 3,4-DHPAA reached maximum levels at 48 h. This suggests that the metabolites of polyphenols derived from microorganisms may have longer biological effects than their parent polyphenols, and that this is determined by differences in the composition of microorganisms in the human intestinal tract [140].

Long-term administration of mixed GTP extract or single EGCG to mice with PD prevented striatal dopamine depletion and substantia nigra pars compacta dopaminergic neuron loss [9,141]. EGCG has been proved to be the most effective free radical scavenger monomer in tea catechins, which may be attributed to the existence of trihydroxyl on the B ring and gallate on the 3′ position of the C ring [68]. Green tea catechins can reduce the production/downregulate pro-apoptotic genes and may slow down the loss of nerve cells [55,68,142]. During aging, imbalance in the intestinal microflora may lead to an increase in gastrointestinal permeability, resulting in an increase in the level of circulating bacterial products, such as muramyl dipeptide [125]. The mucin production of mice decreases with age, resulting in a thinner and discontinuous mucus layer. Mucus is part of the congenital intestinal mucosal barrier, which represents the front line of immune defense by reducing the exposure of antigens and bacteria to intestinal epithelial cells. If the mucus layer is lost, microorganisms may interact with intestinal epithelial cells to cause inflammation [143].

**Figure 3:**
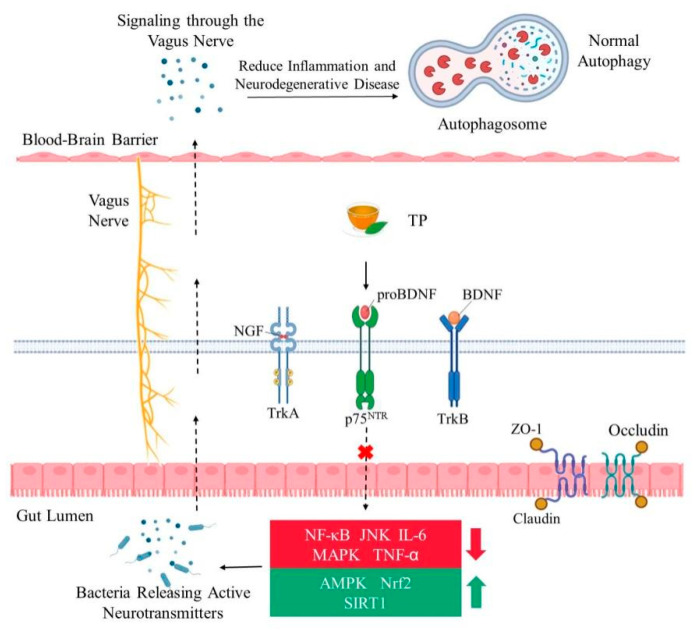
TP maintain intestinal health and inhibit brain neuritis by regulating various signal pathways. TP binds to different neurotrophic factors and transmits signals related to neuronal survival and apoptosis to the inside of the cell, thus regulating cell development and apoptosis. The intervention of TP can inhibit the pro-inflammatory signal pathway and activate anti-inflammatory factors. Under the action of these anti-inflammatory factors, the anti-oxidative stress ability of cells is enhanced. The tight junction proteins are expressed normally, and the intestinal barrier function operates effectively. The intestinal microbiota release beneficial neurotransmitters that are transported along the vagus nerve to the brain. Brain inflammation and neurodegenerative diseases are reduced, and cells can carry out normal autophagy. NGF = nerve growth factor; BDNF = brain-derived neurotrophic factors; TrkA = tropomyosin-related kinase A; p75^NTR^ = p75 neurotrophin receptor; NF-κB = nuclear factor-κB; JNK = c-Jun NH2-terminal kinases; IL-6 = interleukin-6; MAPK = mitogen-activated protein kinase; TNF-α = tumor necrosis factor-α; AMPK = AMP-activated protein kinase; SIRT1 = silent information regulator 1; ZO-1 = zonula occludens-1.

One of the main associated effects of oxidative damage is lipid peroxidation. Polyunsaturated fatty acids are abundant in the lipid bilayers of brain cells. It has been reported that the end products of lipid peroxidation are substantially increased in patients with AD, PD and HD [144]. Increasing evidence indicates that the activation of oxidative stress, the maintenance of low-grade inflammation, and the disturbance of autophagy, are core to biological aging [145]. Recent experiments have demonstrated that TP can improve gut-brain axis dysfunction caused by aging, inhibiting neuroinflammation mediated by TLR4/NF-κB, and counteracting cognitive decline in aging rats [146]. The expression of the tight-junction structure-related proteins zonula occludens (ZO)-1 and occludin decreased in aging mice, and the intervention of TP was observed to reverse this change [147]. Moreover, resultant decrease in lipopolysaccharide and D-lactic acid, which reflect the permeability of rat serum, suggest that TP may effectively maintain the morphology of the intestinal tract [146,148]. There are many indices of intestinal morphology, including villus length, crypt depth, villus/crypt ratio and others. An increase in the ratio of intestinal villus height to the crypt depth can enhance the digestion and absorption functions of animals [149]. The protein expression of TLR4, IL-1 receptor associated kinase and NF-κB p65 in the hippocampus of aging model rats increased, and the expression of inflammatory cytokines IL-1β, IL-6 and tumor necrosis factor TNF-α increased [15]. The intervention of 300 mg/kg TP was able to significantly reduce the expression of these proteins [150]. TP maintain intestinal barrier function mainly by promoting the expression of barrier-forming proteins ZO occludin and claudins, reducing the permeability of the intestinal epithelial barrier and preventing pathogenic factors from redistributing intestinal epithelial connexins in cell membranes and the cytoplasm [11]. TP also reduce the overactivation of microglia by inhibiting the activation of the TLR4/NF-κB inflammatory signal pathway, which may be one of the mechanisms of TP in improving aging-related memory impairment [146] (Figure 3).

Autophagy can prevent cell damage, promote cell survival under nutrient deficiency, and occurs in response to cytotoxic stimuli. Studies have shown that autophagy can play an important role in many physiological and pathological processes, such as cell homeostasis, senescence, immunity, tumorigenesis and neurodegenerative diseases [151]. An increasing number of studies have shown that TP have the potential to promote autophagy [152,153]. When autophagy is normal, the mitochondria involved in protein aggregation, neuroinflammation and dysfunction tend to decrease [16]. The effect of catechin on brain nerve aging has been reported in several animal models. For example, C57BL/6J mice were fed with EGCG for 16 weeks and it was observed that EGCG had a protective effect on insulin resistance and memory impairment induced by a high-fat and high-fructose diet [154]. EGCG significantly promotes lipid metabolism, reduces inflammation and reduces oxidative damage by upregulating IRS-1/AKT and ERK/CREB/BDNF signal pathways to prolong the life of mice [154,155,156]. It was found that EGCG could increase the average lifespan of *Caenorhabditis elegans*; the dose response exhibited an inverted U shape, with the anti-aging activity of EGCG being most marked in the early and middle adult stages [157]. EGCG treatment can prevent apoptosis by reducing the proportion of Bax/Bcl-2 in spinal cord neurons after sciatic nerve crush injury and increasing Trk B and Trk C [78,158,159]. Therefore, the use of green tea catechins, especially EGCG, is a promising treatment for cognitive impairment [79,81,160].

## 6. Conclusions

With increasing age, changes in the intestinal flora are related to the pathogenesis of age-related chronic diseases. Intestinal microbial disorders caused by aging are often accompanied by an increase in intestinal permeability, which causes intestinal microorganisms and their metabolites to enter the blood circulation and cause systemic inflammation. TP is a promising compound for the treatment of age-related chronic low-level inflammatory diseases. Some TP absorbed by the small intestine and their colon metabolites may promote the establishment of probiotics, reduce the production of pathogens, maintain intestinal homeostasis and protect the central nervous system. They can provide potential help in preventing/delaying aging by coordinating multiple signal pathways. At present, the interaction between TP and the intestinal flora provides an improved platform for understanding the specific mechanisms of aging and the promotion of healthy aging to improve the quality of life of the elderly population. There are a range of effective ingredients in different varieties of tea, and their efficacy in the treatment of aging-related neurological diseases is also variable. In the future, the dose-effect relationship of different combinations of the effective components of tea on the intestinal flora and neurodegenerative diseases can be studied in greater depth.

## Figures and Tables

**Table 1:** Tea polyphenols exhibit neuroprotective effects—in vivo and in vitro evidence.

Research Environment	Model Used	Neuroprotective Effects	Reference
In vivo	Sprague–Dawley male rats	Inhibition of PI3K/AKT/mTOR pathway and upregulate autophagy process	[15]
	C57BL/6J	Enhance memory and extend lifespan	[59]
	APPsw transgenic mice	Aβ and plaques levels decreased	[16]
	Male Long–Evans rats	Inhibits MAO-B enzyme activity in rat brain	[78]
	ICR mice	Enhance spatial memory, inhibit neuronal damage in the brain	[79]
	Human	Improve brain compensatory response and cognitive reserve ability	[70]
In vitro	U118MG cells	Increase BDNF gene expression significantly	[2]
	Human neuroblastoma SH-SY5Y cells	Inhibit neuronal cell death caused by the neurotoxins 6-hydroxydopamine (6-OHDA) and 1-methyl-4 phenylpyridinium (MPP^+^)	[9]
	PC12 cells	Inhibit the aggregation of α-synuclein	[17]
	PC12 cells	Reduce cell death and promote neurite outgrowth	[80]
	BV2 microglial cells	Inhibit LPS-induced inflammation, reduce TNF-α secretion, iNOS and COX-2 protein expression	[79,81]

## Data Availability

Not applicable.
